# Factors related to preventive COVID-19 behaviors using health belief model among general population: a cross-sectional study in Iran

**DOI:** 10.1186/s12889-021-11983-3

**Published:** 2021-10-24

**Authors:** Mahmood Karimy, Fatemeh Bastami, Robab Sharifat, Akbar Babaei Heydarabadi, Naser Hatamzadeh, Amir H. Pakpour, Bahman Cheraghian, Fereshteh Zamani-Alavijeh, Mehrnoosh Jasemzadeh, Marzieh Araban

**Affiliations:** 1 Social Determinants of Health Research Center, Saveh University of Medical Sciences, Saveh, Iran; 2 Department of Public Health, Faculty of Health and Nutrition, Lorestan University of Medical Sciences, Khorramabad, Iran; 3 Omidyeh Health Care System, Ahvaz Jundishapur University of Medical Sciences, Ahvaz, Iran; 4 Department of Health Education and Promotion, School of Public Health, Ahvaz Jundishapur University of Medical Sciences, Ahvaz, Iran; 5 Department of Nursing, School of Health and Welfare, Jönköping University, Jönköping, Sweden; 6 Social Determinants of Health Research Center, Research Institute for Prevention of Non-Communicable Diseases, Qazvin University of Medical Sciences, Qazvin, Iran; 7 Department of Biostatistics and Epidemiology, School of Public Health, Ahvaz Jundishapur University of Medical Sciences, Ahvaz, Iran; 8 Department of health education and promotion, School of Health, Isfahan University of Medical Sciences, Isfahan, Iran

**Keywords:** COVID-19, Preventive behavior, Health belief model, Iran

## Abstract

**Background:**

Coronavirus disease (COVID-19) pandemic has become one of the biggest challenges to global health and economy. The present study aimed to explore the factors related to preventive health behaviors during the COVID-19 pandemic in Khuzestan Province, South of Iran, using the Health Belief Model (HBM).

**Methods:**

The present cross-sectional study was conducted in the period between July 2020 and September 2020. A total of 1090 people from Khuzestan province participated in the study. The data collection method included a multistage cluster sampling method with a random selection of provincial of health centers. The questionnaire collected socio-demographic information and HBM constructs (e.g., perceived susceptibility, perceived severity, perceived benefits and barriers, cues to action, and COVID-19 preventive behaviors). Data were analyzed using ANOVA, t-test, hierarchical multiple linear regression, and SPSS version 22.

**Results:**

The mean age of the participants was 35.53 ± 11.53, more than half of them were female (61.6%) and married (65.3). The results showed that 27% of the variance in the COVID-19 preventive behaviors was explained by HBM constructs. The regression analysis indicated that female gender (β = 0.11), perceived benefits (β = 0.10), perceived barriers (β = − 0.18), external cues to action (β = 0.25), and internal cues to action (β = 0.12) were significantly associated with COVID-19 preventive behaviors (*p* < 0.05).

**Conclusion:**

Designing an educational intervention on the basis of HBM might be considered as a framework for the correction of beliefs and adherence to COVID-19 behavior. Health information campaigns need to (1) emphasize the benefits of preventive behaviors including avoiding the likelihood of getting a chronic disease and complications of the disease, (2) highlight the tips and advice to overcome the barriers (3) provide cues to action by means of showing various reminders in social media (4) focusing on adoption of COVID-19-related preventive behaviors, especially among men.

**Supplementary Information:**

The online version contains supplementary material available at 10.1186/s12889-021-11983-3.

## Background

The coronavirus disease 2019 (COVID-19) pneumonia pandemic is a new emerging global challenge in the management of infectious diseases [1–4]. At present, the disease has spread rapidly and affected the whole world with epidemiological features such as rapid transmission, increasing prevalence in a short period of time, extensive incubation period (2–14 days), as well as the ability to infect all individuals and groups [5].

A number of measures have been developed to prevent and mitigate the transmission and mortality associated with COVID-19 including rapid identification of suspected cases, rapid testing and isolation, contact tracing and quarantine, restrictions on non-essential domestic and international travel, and high-level regular participation of communities [6]. Currently, the transmission routes of COVID-19 infection are well recognized. Governments and other sectors such as media, health workers, celebrities, police, and other stakeholders have focused primarily on behaviors that prevent the transmission of the virus, such as wearing masks, keeping physical distance, hand-washing practices, and avoiding public meetings (such as religious ceremonies and family gatherings) [7, 8]. Despite taking preventive programs, the rate of COVID-19 infection remains high, indicating that these programs have not been effective in controlling the infection [9, 10]. For instance, a study by Smith et al. [11] among adolescents aged 12–15 years in 80 countries showed that the prevalence of never/rarely hand washing practices before eating and after using the toilet was 6.4, and 5.6%, respectively.

In Iran, previous studies have shown that despite the recommendations of international, national, and regional organizations, the level of behavioral adherence to prevent coronavirus in the general population is still unsatisfactory [9]. Therefore, it is necessary to identify factors affecting adherence to recommended guidelines. Behavioral models are designed to identify factors that influence behavior change in order to control the infection [12]. Various models/theories have been proposed to identify factors affecting behavior change. The Health belief model (HBM) is a comprehensive model in health-behavioral sciences [13]. Previous studies have supported the HBM as a useful model for predicting/explaining preventive behaviors against infectious diseases such as COVID-19 [8, 13].

HBM is a model that focuses on individual beliefs about health conditions [7]. According to this model, the probability that a person participates in a health practice is based on individual beliefs; the probability of adopting recommended behaviors (e.g. preventive COVID-19 behaviors) will increase by changing the individual perceptions. According to the health belief model, people will adopt preventive health behavior when they feel threatened by pandemic situations (perceived susceptibility) or consider that the disease can have serious ramifications to their health (perceived severity). Likewise, with the information and guidance people receive from their surroundings or inner environment (cues to action), they believe in the usefulness of preventive behaviors such as using a mask (perceived benefits), and the perception of negative aspects (costs) of a given behavior to perform (perceived barriers) [13, 14].

According to the World Health Organization (WHO) report (June 06, 2021), Iran had 2,966,363 COVID-19 cases [15]. Khuzestan province, southwest of Iran, is among the regions with the highest number of COVID-19 cases [16]. Considering the importance of preventive behaviors in reducing the transmission of COVID-19, the present study aimed to explore the factors related to preventive health behaviors during the COVID-19 pandemic in Khuzestan province using the Health Belief Model (HBM).

## Methods

### Study design and participants

The present cross-sectional study was conducted in the period between July 2020 and September 2020. A total of 1090 citizens from the catchment area of Khuzestan province participated in the study. The inclusion criteria were as follows:15 years of age or older, ability to communicate in Persian, living in Khuzestan province, and voluntary consent to participate in this study.

### Sampling

A multistage cluster sampling method was adopted for the recruitment of study participants. The counties of Khuzestan province, Ahvaz County (the capital of Khuzestan province) and five other counties (clusters) including Omidiyeh, Izeh, Shush, Hoveyzeh, and Dasht-e Azadegan were selected as a cluster unit. Within each county (cluster) there were two elements (urban and rural). For sampling of urban areas, a health center from the downtown area and one health center from the south of city were randomly selected using a map of the population area. In rural areas, the potential list of the population was prepared by a health care provider who was not a member of the study group. Then, a systematic random sampling technique with a sampling interval of three was employed to select the study participants. The selected geographic location in the map was not limited to any specific geographic region. Then, within the selected urban and rural health centers, based on the file number of households, systematic sampling with probability proportional to size was applied to select the households, i.e., the larger health center population density, the higher the share of the total sample size. In the final stage, two individuals (preferably a man and a woman) were sampled within each household. “The final sample was 1,090”: (Ahvaz = 425, Omidieh =369, Izeh = 86, Shush = 107, Hoveyzeh = 71, and Dasht-e Azadegan = 32).

### Measures

A three-part self-report -researcher-made- questionnaire (Additional file 1) which relied on demographic, HBM constructs, and preventive behaviors items was used to collect data.

### Demographic sheet

The first section of the questionnaire included demographic characteristics including age, sex, education, occupation, and history of chronic diseases.

### HBM constructs

The second section of the questionnaire included questions on HBM structures as follows: 1- Perceived susceptibility (7 items); 2- Perceived severity (5 items); 3- Perceived benefit (4 items); 4- Perceived barrier (5 items); 5- Internal cues to action (4 items); and 6- External cues to action (4 items). All items were rated on a five-point Likert type scale ranging from strongly disagree (1) to strongly agree (5), (Table 1).

**Table 1:** Description of the HBM constructs

HBM constructs	number of items	score ranges	Cronbach’s alpha	McDonald’s ω
Perceived susceptibility	7	7–35	0.72	0.78
Perceived severity	5	5–25	0.70	0.72
Perceived benefits	4	4–20	0.78	0.78
Perceived barriers	5	5–25	0.85	0.86
internal cues to action	4	4–20	0.75	0.75
External cues to action	4	4–20	0.87	0.87

### Preventive behaviors

The third part of the questionnaire included questions concerning the preventive COVID-19 behavior of the individual during the last month. This part was designed based on similar studies [17–20]. Seven items were used to measure preventive COVID-19 behavior: The behavior questions included self-report items such as: Do you wash your hands for 20 s after touching surfaces or outdoor items? Do you use a mask in public places? Do you refuse to go to family ceremonies (celebrations, funerals, parties, etc.)? Do you cover your mouth when sneezing or coughing? Do you disinfect surfaces with disinfectants? Do you adhere to the principles of social distancing? Do you disinfect commonly used surfaces and equipment with disinfectants? The rating scale was on a five-point scale: never = 1, rarely = 2, sometimes = 3, often = 4, always = 5. The possible range of scores was 7–35. The internal consistency was acceptable as α = 0.89.

### Validity & reliability

The validity of the present questionnaire was determined by face and content validity methods. The face validity of the items (quantitative and qualitative (was determined by 20 participants and calculated using the impact score equation) Impact Score = Frequency (%) × Importance). Questions with an impact score higher than 1.5 were considered acceptable. At this stage, two questions were deleted. The qualitative content validity of this questionnaire was observed and evaluated by 10 infectious disease and public health specialists. The content validity ratio (CVR) was determined using the Lawshe table (CVR values of higher than 0.75). The specialists were enquired to stipulate whether an item is necessary for running a construct in a set of items. The specialists were requested to rate each item according to the Content Validity Index (CVI), relevance, clarity, and simplicity (CVI value 0.79 was considered acceptable) [21]. The internal consistency reliability of all constructs was found to be satisfactory (α > 0.70, Table 1).

### Sample size

The sample size was determined according to the following formula based on the data obtained from a pilot study in which the smallest correlation was considered the largest sample size. $\mathrm{n}={\left[\frac{\mathrm{Z}1-\upalpha /2+\mathrm{Z}1-\mathrm{B}\ }{0.5\ \mathrm{Ln}\left(\ \frac{1+r}{1-r}\right)}\right]}^2+3$; d = 0.05, β = 0.2, *r* = 0.12. Finally, the sample size was estimated to be 540 people. The final sample size was determined to be 1080 people (considering design effect equal to 2). A total of 1100 questionnaire sheets were distributed to the participants to obtain the required 1080.

#### Data analysis

Inferential statistics (independent t-test and one-way analysis of variance) were used for comparisons between groups. Correlation and multiple hierarchical linear regressions were performed to test the relationships between HBM and the health-related behaviors. To explore the factors affecting preventive behaviors, bivariate analysis was performed with demographic variables and HBM constructs, of which, gender, education (the number of years of formal education), occupation, marital status, and HBM constructs were significantly associated with behavior. In the next step, variables associated with the outcome in the bivariate analysis with *p*-value < 0.25 were entered in the model. An analysis of residuals confirmed the assumptions of linearity. It should be mentioned that collinearity was checked and was negative. Data were analyzed using SPSS software Version 22.0 (SPSS Inc., Chicago, IL, USA). *P*-value less than 0.05 at the final stage was considered statistically significant.

### Ethical considerations

The study was approved by the Ethics Committee of Ahvaz Jundishapur University of Medical Sciences, Ahvaz, Iran (Registration No: IR.AJUMS.REC.1399.145). Written informed consent was obtained from the participants. During data collection, social distancing was maintained and in the period of collecting the responses, both the respondent and the research assistants were wearing face masks.

## Results

Among the 1100 distributed questionnaires, 10 questionnaires were excluded from the analysis owing to incomplete and distorted information.

The mean age of the participants was 35.53 ± 11.53 years, the majority of them were women (61.6%) and married (65.3%). The participants’ level of education ranged from high school (36.8%) to university education (30.8%). In the case of men, 31.9% were self-employed and 29.3% were employees. The highest percentage of occupation in women was 66.6% (homemaker (, 19.3% (Employee), and 7.3% (self-employment), (Table 2).

**Table 2:** Descriptive statistics of demographic variables

Variables		
**Age**	**N**	**%**
**15–29**	320	29.4
**30–49**	530	48.6
**50–69**	172	15.7
**≥ 70**	68	6.2
**Gender**		
**Male**	419	38.5
**Female**	671	61.5
**Education**		
**University**	336	30.8
**High school**	401	36.8
**Secondary school**	270	24.7
**Primary school**	83	7.6
**Marital status**		
**Married**	712	65.3
**Divorced or widowed**	121	11.1
**Single**	257	23.6
**Male occupation**		
**Worker**	107	25.5
**Employee**	123	29.3
**Self-employed**	134	31.9
**Retired**	39	9.3
**Jobless**	16	3.8
**Female occupation**		
**Housewife**	447	66.6
**Worker**	33	4.9
**Employee**	130	19.3
**self-employment**	49	7.3
**Retired**	12	1.2
**History of chronic disease**		
**Yes**	258	23.7
**No**	832	76.3

More than two-thirds of the participants (76.3%) reported that they had no chronic disease. Hypertension (5.5%) and diabetes (5%) were the most prevalent self-reported doctor-diagnosed chronic conditions.

There was a positive and significant correlation between HBM constructs and COVID-19 preventive behaviors (external cues to action (*r* = .47), internal cues to action (*r* = .42), perceived benefits (*r* = .30), and perceived severity (*r* = .21). A negative correlation was found between perceived barriers (*r* = −.20) and COVID-19 preventive behaviors (*p* < 0.05, Table 3).

**Table 3:** Correlations between behavior and HBM construct

	Perceived susceptibility	Perceived severity	Benefit	Barrier	Internal Cues to action	External Cues to action	behavior
**Perceived susceptibility**	1						
**Perceived severity**	0.31^b^	1					
**Benefit**	0.03	0.23^b^	1				
**Barrier**	0.65^b^	0.16^a^	−0.08^a^	1			
**Internal Cues to action**	0.07^a^	0.26^b^	0.31^b^	−0.03	1		
**External Cues to action**	0.02	0.27^b^	0.38^b^	−0.13^b^	0.59^a^	1	
**behavior**	0.04	0.21^b^	0.30^b^	−0.20^a^	0.42^b^	0.47^b^	1

^a^Correlation is significant at the 0.01 level (2-tailed).

^b^Correlation is significant at the 0.05 level (2-tailed)

Table 4 shows that the perceived severity of COVID-19 among older people (aged + 70 years) was more than that of younger people (aged 15–29). A significant correlation was found between constructs of cues to action and preventive behaviors (p < 0.05). Also, women had better preventive behaviors than men. Similarly, people with university-level education compared to the elementary level, married people compared to single people, and people without any chronic disease had better preventive behaviors to COVID-19. Employees’ occupational group had better mean scores of preventive behaviors than workers and unemployed people (p < 0.05, Table 4).

**Table 4:** Comparisons of mean scores for the HBM constructs, and preventive behaviors across demographic variables

	Perceived susceptibility	Perceived severity	Benefit	Barrier	Internal Cues to action	External Cues to action	behavior
**Age**	M ± SD	M ± SD	M ± SD	M ± SD	M ± SD	M ± SD	**M ± SD**
15–29	21.2 ± 5.5	18.5 ± 5.2	16.4 ± 3.2	14.0 ± 5.1	15.8 ± 4.2	16.0 ± 3.3	**30.8 ± 5.1**
30–49	21.0 ± 5.2	19.5 ± 4.3	17.2 ± 3.2	13.4 ± 5.6	16.5 ± 3.2	17.1 ± 2.9	**32.0 ± 4.1**
50–69	21.7 ± 5.0	19.7 ± 6.2	17.1 ± 3.9	15.2 ± 5.1	15.8 ± 3.3	16.5 ± 3.0	**31.4 ± 4.2**
≥ 70	22.2 ± 6.4	19.0 ± 2.1	16.7 ± 2.4	13.6 ± 5.0	16.6 ± 2.2	16.5 ± 3.1	**33.6 ± 3.1**
*P* value (derived from ANOVA)	0.63	0.022	0.005	0.01	0.03	0.001	**0.007**
**Gender**							
Male	21.4 ± 5.2	19.8 ± 5.7	17.0 ± 3.2	14.1 ± 5.4	16.2 ± 3.2	16.7 ± 3.0	**31.2 ± 4.5**
Female	21.0 ± 5.2	18.8 ± 4.6	16.8 ± 3.4	13.3 ± 5.2	16.2 ± 3.8	16.7 ± 3.1	**31.9 ± 4.4**
*P* value (derived from t test)	0.29	0.001	0.31	0.02	0.91	0.90	**0.02**
**Education**							
University	19.8 ± 5.0	19.1 ± 3.9	17.5 ± 3.0	12.2 ± 5.2	16.7 ± 4.0	17.2 ± 2.9	**32.2 ± 3.5**
High school	20.9 ± 4.8	19.0 ± 5.1	16.9 ± 3.0	13.6 ± 5.3	16.1 ± 3.5	16.7 ± 3.1	**31.7 ± 4.7**
Secondary school	23.0 ± 6.1	19.7 ± 5.7	16.5 ± 3.9	15.2 ± 5.5	15.6 ± 3.4	16.2 ± 3.2	**31.2 ± 4.6**
Primary school	24.0 ± 4.5	19.3 ± 3.8	16.5 ± 3.0	16.0 ± 4.9	15.7 ± 2.7	15.8 ± 2.5	**29.5 ± 5.1**
*P* value (derived from ANOVA)	0.001	0.163	0.001	0.001	0.01	0.001	**0.001**
**Marital status**							
Married	23.1 ± 7.0	19.6 ± 4.8	17.1 ± 3.2	13.1 ± 5.3	16.3 ± 3.3	15.8 ± 3.5	**32.2 ± 4.0**
Divorced or widowed	23.5 ± 7.1	17.9 ± 4.5	15.9 ± 2.9	14.8 ± 5.1	15.6 ± 3.1	17.1 ± 3.0	**30.1 ± 5.7**
Single	21.6 ± 6.5	18.1 ± 4.4	16.4 ± 3.4	14.3 ± 5.0	15.7 ± 4.5	15.9 ± 3.2	**30.3 ± 5.3**
*P* value (derived from ANOVA)	0.001	0.001	0.001	0.002	0.001	0.001	**0.001**
**Occupation**							
housewife	21.7 ± 5.4	19.2 ± 4.5	16.6 ± 3.4	14.1 ± 5.1	16.0 ± 3.3	16.6 ± 2.9	**31.9 ± 4.1**
worker	21.9 ± 4.9	19.8 ± 3.7	16.6 ± 3.3	15.2 ± 5.9	16.1 ± 3.7	16.4 ± 3.4	**30.5 ± 5.3**
Employee	20.1 ± 4.5	18.9 ± 4.6	17.7 ± 3.1	11.9 ± 5.0	16.7 ± 3.3	17.2 ± 3.0	**32.4 ± 3.8**
self-employment	21.1 ± 5.3	19.9 ± 7.5	16.7 ± 3.4	13.6 ± 5.5	15.9 ± 3.1	16.5 ± 3.0	**31.3 ± 4.6**
Retired	21.8 ± 7.0	19.5 ± 4.7	16.6 ± 2.4	15.6 ± 5.5	16.7 ± 2.9	17.4 ± 3.3	**31.2 ± 4.6**
workless	21.8 ± 5.4	19.6 ± 3.5	15.4 ± 3.2	16.3 ± 4.4	14.3 ± 2.1	14.0 ± 2.3	**27.3 ± 6.0**
*P* value (derived from ANOVA)	0.001	0.001	0.001	0.001	0.001	0.001	**0.001**
Chronic disease							
Yes	21.5 ± 5.4	19.1 ± 4.9	17.2 ± 3.0	14.1 ± 5.8	16.2 ± 3.6	16.7 ± 3.2	**31.3 ± 4.5**
No	21.1 ± 5.2	19.6 ± 5.4	16.8 ± 3.2	13.5 ± 5.2	16.1 ± 3.7	16.8 ± 3.0	**31.7 ± 4.3**
*P* value (derived from t test)	0.44	0.223	0.15	0.26	0.90	0.89	**0.21**

The regression analysis indicated that female gender (β = 0.11), perceived benefits (β = 0.10), perceived barriers (β = − 0.18), external cues to action (β = 0.25), and internal cues to action (β = 0.12) were significantly associated with COVID-19 preventive behaviors (p < 0.05), (Table 5).

**Table 5:** Multiple hierarchical linear regression analysis for the assessment of health beliefs and behavior

	Un standardized Coefficients		Standardized β	t	95%confidence interval for B		***P*** Value
	B	SE			Lower Bound	Upper Bound	
Age	0.023	0.017	0.056	1.363	−0.010	0.057	0.173
Gender (female)	1.038	0.355	0.115	2.923	0.340	1.735	0.004
Married	0.647	0.390	0.066	1.660	−0.119	1.413	0.098
Education	0.080	0.053	0.062	1.518	−0.024	0.184	0.130
Perceived benefit	0.129	0.051	0.105	2.562	0.030	0.229	0.011
Perceived barriers	−0.155	0.04	− 0.182	−3.910	− 0.233	−0.077	< 0.001
Perceived severity	0.080	0.044	0.079	1.837	−0.006	0.166	0.067
External Cues to action	0.348	0.068	0.252	5.094	0.214	0.482	< 0.001
Internal Cues to action	0.158	0.060	0.129	2.639	0.040	0.275	0.009
Perceived susceptibility	0.042	0.043	0.047	0.985	−0.042	0.127	0.325

**Model Adjusted R Square=0.268**

## Discussion

The Health Belief Model (HBM) proposes that the person’s attitude influences health-related behavior and perception, i.e. changing attitudes and beliefs lead to preventive health behavior [22]. The results of the present study showed a significant positive correlation among internal/external cues to action, perceived severity and benefits, and COVID-19 preventive behaviors. Furthermore, a negative correlation was found between perceived barriers and COVID-19 preventive behaviors. In general, the present study results were in line with the results of previous studies [18, 23–26].

The present study showed that COVID-19 perceived severity in older people (aged 70+) was more than that of younger people (aged 15–29). Studies have shown that older people are at higher risk of COVID-19. Similarly, national clinical COVID-19 registries have shown a higher mortality rate among the elderly [27–29]. Jiang et al. suggested that the perceived threat to SARS preventive behavior was an important predictor of behavior change [30]. People who believe they are at high risk for the disease are more likely to engage in preventive behaviors. Therefore, educational programs should promote the perceived susceptibility and severity of COVID-19. Clark et al., 2020 concluded that age was not generally associated with voluntary compliance behaviors which was inconsistent with the results of the present study [31]. This study also indicated that women had better preventive behaviors than men and people with university-level compared to the elementary level, married people compared to single people, and people without the underlying disease had better preventive behaviors to COVID-19. Similarly, Clark et al., 2020 found that women were more likely to engage in health-related behaviors than men which was consistent with the results of the previous studies [32, 33].

The results of this study suggested that HBM almost described 27% of the variance (adjusted R2) in the COVID-19 preventive behaviors. These results are consistent with some previous studies. Mirzaei et al. identified that HBM explained 29.2% of the variance of preventive behaviors of COVID-19 [34]. In another study by Fathian-Dastgerdi, HBM constructs explained 42% of the variance of preventive behaviors [35]. Wang et al. in a study conducted in the wake of the COVID-19 pandemic in China showed that the strongest predictor of behavioral change was perceived susceptibility [1]. This may be explained due to the fact that during the early stage of the pandemic there was a more precautionary motive to take preventive actions. The present study was conducted a few months after the outbreak of COVID-19 when the level of public awareness about disease prevention had increased.

In the present study, internal/external cues to action were among the strongest stimulus needed to trigger preventive behavior, i.e., promoting the adoption of preventive behaviors by mass media, emphasizing on the sense of social responsibility, and sending notifications via cellphone and social media could promote COVID-19 preventive behaviors.

The present study, also showed that perceived barriers were an essential factor that determines behavioral change. Perceived barriers were considered as an important factor for preventive behaviors. So, focusing (identifying, eliminating) on the barriers which hinder COVID-19 preparedness and response in humanitarian settings is necessary [22].

Currently, no licensed antiviral drug has been approved for the treatment of COVID-19. Quarantine (staying home) and social distancing policy have become strategic in controlling the COVID-19 pandemic. However, during quarantine, some people do not meet the health protocols requirements for various reasons. For people who violate a stay-at-home order, supportive community measures should be taken and taught. Furthermore, a more dynamic and vibrant environment at home which increases people’s resilience during lockdown should be provided [36–38].

### Strengths and limitations of the study

The cross-sectional nature of the study makes it challenging to derive causal relationships. The results might not be generalizable to all Iranian population. The results of the study might be subjected to recall bias as the participants were required to answer behavior questions over the past month.

Multi-stage and random sampling methods, trained interviewers, reliable and valid Health Belief model questionnaire, were among the strengths of the study.

## Conclusion

Designing an educational intervention on the basis of HBM might be considered as a framework for the correction of beliefs and adherence to COVID-19 behavior. Health information campaigns need to (1) emphasize the benefits of preventive behaviors including avoiding the likelihood of getting a chronic disease and complications of the disease, (2) highlight the tips and advice to overcome the barriers (3) provide cues to action by means of showing various reminders in social media (4) focusing on adoption of COVID-19-related preventive behaviors, especially among men.

## Supplementary Information

**Additional file 1.** The questionnire used in the study to collect the data. The first part of the questionnaire included demographic characteristics. The second part of the questionnaire consisted of HBM constructs. The third part consisted of behaviors.

## Data Availability

The datasets used and/or analyzed during the current study are available from the corresponding author on reasonable request.

## Abbreviations

HBM: Health Belief Model
COVID-19: Coronavirus disease 2019
