# Supplementary material for: Factors related to preventive COVID-19 behaviors using health belief model among general population: a cross-sectional study in Iran
Source: BMC Public Health. 2021 Oct 24;21:1934. doi: 10.1186/s12889-021-11983-3 (PMC8542411; doi:10.1186/s12889-021-11983-3)
Supplement: Supplementary file 1 — Additional file 1. The questionnire used in the study to collect the data. The first part of the questionnaire included demographic characteristics. The second part of the questionnaire consisted of HBM constructs. The third part consisted of behaviors. [file 12889_2021_11983_MOESM1_ESM.doc]

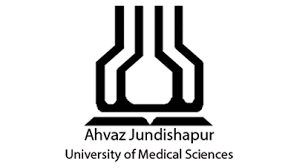


***Additional file 1***

Title of data: The questionnire used in the study to collect the data

**Date:** ......../......../..........

**Questionnaire code:** .................... **Name of the Health center:** ...............................

| **Dear, Respondent**  The present questionnaire is a part of research project entitled “Investigating Preventive Behaviors Toward COVID-19”. The questionnaire is designed to assess your beliefs and practice towards Covid-19. The results of the questionnaire will be beneficial for health care providers. Similarly, the results of the questionnaire will encourage the health promotion and disease prevention in community settings during Covid-19 pandemic. Please read each question carefully and answer as accurately as you can. It is noteworthy that the answers may be influenced by the respondents' experiences with the survey topic. There are no right or wrong answers, so please choose the survey responses that best describe your own experience. The questionnaire is anonymous and your responses will be kept strictly confidential.  **Ethics ID: IR.AJUMS.REC.1399.145** |
| --- |

**Part A.** Demographic information

**Q1. Age: ……………….……...**  **Q2**. **Gender:** Male **5** Female **5**

**Q3. Marital status:** Single**🖵** Married**5** Widowed**5** Divorced**5**

**Q4. Education Level: ………………………………….…**

**Q5. Years of schooling** (The average number of years spent in school): ………………………………….…

**Q6. Employment status**: Housewife 5 Worker 5 Self-employed5 Employee5

In school5 Retired5 Others 5 (List others……………………………………...……….……)

**Q7. The average monthly family income?**

1. Low (living in poor households) 5
2. Medium (Less than necessary to cover all living expenses) 5
3. Good (Can afford to cover all living expenses) 5
4. Excellent (More than necessary to cover all the cost of living) 5

**Q8. Do you have a history of any disease?** Yes 5 No5

**Please indicate if you have a history of the following:**

Diabetes 5 Cardiovascular disease 5 Blood pressure 5 Lung disease 5

Kidney disease 5 Cancer 5 Other5(List others……………………………………...……….……)

**Q9. Which of the following places have you been to in the last two weeks? (**You can choose more than one option**).**

Public places 5 Ceremonies (celebrations, funerals, ...) 5

Close contact with someone with COVID-19 5 Health centers 5

**Q10. What is the most common reason for not observing quarantine rules and the lockdown?**

Jobs and income provision 5 Shopping for basic necessities 5 Fatigue from staying at home 5

Adherence to ethnic customs 5 Growing distrust of COVID-19 News 5

Distrust in doctors and healthcare workers 5 Hesitancy or distrust in complications and death from COVID-​195

**Part B. Please respond to the following questions by placing a check mark (√) in the answer box**

| Please read each question carefully and answer as accurately as you can. | **Strongly Agree** | **Agree** | **Neutral** | **Disagree** | **Strongly**  **Disagree** |
| --- | --- | --- | --- | --- | --- |
| **Category** |
| I'm afraid of the Coronavirus disease |  |  |  |  |  |
| I feel uncomfortable when I think about the Coronavirus disease |  |  |  |  |  |
| When I think about the Coronavirus disease, I feel a sudden cold and wet on my hand. |  |  |  |  |  |
| I am afraid of losing my life due to the Coronavirus disease |  |  |  |  |  |
| I feel more anxious and worried when watching the news and stories about the Coronavirus disease on social media |  |  |  |  |  |
| I can't even sleep comfortably because I might get the Coronavirus disease |  |  |  |  |  |
| When I think that I might get the Coronavirus disease, my heart rate (heart rhythm) rises sharply (I feel like it's pounding, fluttering or beating irregularly) |  |  |  |  |  |

**Part C. Please respond to the following questions by placing a check mark (√) in the answer box**

| *Please* read each question carefully and answer as accurately as you can. | **Strongly Agree** | **Agree** | **Neutral** | **Disagree** | **Strongly**  **Disagree** |
| --- | --- | --- | --- | --- | --- |
| **Category/Question** |
| **Perceived Susceptibility** |
| I am strong enough to fight off the Coronavirus disease |  |  |  |  |  |
| I may be infected with the Coronavirus disease, but apparently, I don't have a symptom. |  |  |  |  |  |
| I may also be infected with the Coronavirus disease |  |  |  |  |  |
| In my opinion, personal hygiene should only be observed when people are at increased risk of infection. |  |  |  |  |  |
| I think that the risk of the Coronavirus infection has been exaggerated by health care providers |  |  |  |  |  |
| When I have no problems, it means I am healthy and do not need to follow healthcare recommendation. |  |  |  |  |  |
| It can be upsetting to hear about the crisis and see images from the Coronavirus disease. |  |  |  |  |  |
| **Perceived Severity** |  |  |  |  |  |
| If I have the Coronavirus disease. I may have a severe lung infection (Pneumonia) |  |  |  |  |  |
| If I start feeling sick and think I may have the Coronavirus disease, It is possible to transmit the virus to my family and relatives. |  |  |  |  |  |
| I may be hospitalized for a long time due to the Coronavirus disease |  |  |  |  |  |
| My body/ immune system is strong and it is not possible to experience medical or mental health problem |  |  |  |  |  |
| A lapse in COVID-19 control can cost me too much |  |  |  |  |  |
| I may even die because of the Coronavirus disease |  |  |  |  |  |

**Part D. Please respond to the following questions by placing a check mark (√) in the answer box**

| Please read each question carefully and answer as accurately as you can. | **Strongly Agree** | **Agree** | **Neutral** | **Disagree** | **Strongly**  **Disagree** |
| --- | --- | --- | --- | --- | --- |
| **Category/Question** |
| **Perceived Barriers** |
| Due to the shortage of masks, I can't wear masks regularly. |  |  |  |  |  |
| I can't wash my hands regularly because of boredom |  |  |  |  |  |
| Due to the high cost of the mask, I cannot afford it. |  |  |  |  |  |
| I don't believe in hand washing and masks wearing for infection prevention and control |  |  |  |  |  |
| I know that everything (Life, Health, and Disease) is in God sovereign control. So, I cannot do much to protect myself and others from the Coronavirus disease |  |  |  |  |  |
| **Perceived benefits** |  |  |  |  |  |
| If I continue to follow the guidance from healthcare providers, I will be safe against the Coronavirus disease |  |  |  |  |  |
| According to the health team's recommendations, if I do individual protection, I have contributed to the health of my family and community. |  |  |  |  |  |
| A timely visit to a doctor or health centers will protect me from the complications of the Coronavirus disease and subsequent problems. |  |  |  |  |  |
| The cost of prevention (e.g., using a mask/regular visit to a doctor) is lower than the cost of treatment. |  |  |  |  |  |
| Self-efficacy |  |  |  |  |  |
| I can wash my hands several times a day and at least 20 seconds at a time even if I'm not in the mood. |  |  |  |  |  |
| I can get masks even if it's expensive. |  |  |  |  |  |
| I can visit a doctor to diagnose the symptoms of the Coronavirus, even if it costs me too much |  |  |  |  |  |
| I can take care of myself against the Coronavirus even if the people around me don't remind me |  |  |  |  |  |
| I can prepare and consume healthy food even if it requires a lot of time and patience. |  |  |  |  |  |
| I can follow self-care recommendations, even if it's time-consuming, costly, or.... |  |  |  |  |  |
| I cannot participate in family ceremonies (celebration, funeral ...) despite the expectations of those around me and my relatives. |  |  |  |  |  |
| Despite my fatigue, impatience, and inability, I can take regular personal protective measures. |  |  |  |  |  |

**Part E. Please respond to the following questions by placing a check mark (√) in the answer box**

| Please read each question carefully and answer as accurately as you can. | **Strongly Agree** | **Agree** | **Neutral** | **Disagree** | **Strongly**  **Disagree** |
| --- | --- | --- | --- | --- | --- |
| **Category/Question** |
| *'***External***'* **cues to action** |
| In my opinion, radio and TV broadcasting can encourage me to follow and observe health recommendation |  |  |  |  |  |
| In my opinion, the recommendations of the healthcare providers can encourage me to follow and observe health recommendation. |  |  |  |  |  |
| In my opinion, my family and relatives can encourage me to follow and observe health recommendation. |  |  |  |  |  |
| In my opinion, the Internet and mobile can encourage me to follow and observe health recommendation. |  |  |  |  |  |
| '**Internal**' **cues to action** |  |  |  |  |  |
| Fear of possible exposure to COVID-19 encourages me to follow and observe health recommendation. |  |  |  |  |  |
| Fear and fatigue of quarantine and isolation from family and society due to COVID-19 encourages me to follow and observe health recommendation. |  |  |  |  |  |
| Fear of death encourages me to follow and observe health recommendation |  |  |  |  |  |
| A sense of social responsibility encourages me to follow and observe health recommendation. |  |  |  |  |  |

**Part F. Please respond to the following questions by placing a check mark (√) in the answer box**

| Please read each question carefully and answer as accurately as you can. | **Always** | **Often** | **Sometimes** | **Rarely** | **Never** |
| --- | --- | --- | --- | --- | --- |
| **Category/Question** |
| **Behavior questionnaire** |
| 1- Do you wash your hands for 20 seconds after touching surfaces or outdoors? |  |  |  |  |  |
| 2- Do you cover your mouth when sneezing or coughing? |  |  |  |  |  |
| 3- Do you refuse handshakes, social kissing, and greetings during the COVID-19 pandemic. |  |  |  |  |  |
| 4- Do you wear masks in public places? |  |  |  |  |  |
| 5- Do you clean/disinfect surfaces with disinfectants? |  |  |  |  |  |
| 6- Do you adhere to the principles of social distancing? |  |  |  |  |  |
| 7- Do you refuse to go to family gatherings/events (celebrations, funerals, parties,…). |  |  |  |  |  |

We are extremely grateful for your contributing your valuable time, honest information, and thoughtful suggestions in identifying people's health education needs. If you wish, write down your email or phone number so that we will share these results with you after completion of the research.

**Tel:** **Email:**

Sincerely,

Dr. Marzieh Araban
